# Effect of Cytochrome P450 Family 2 Subfamily R Member 1 Variants on the Predisposition of Coronary Heart Disease in the Chinese Han Population

**DOI:** 10.3389/fcvm.2021.652729

**Published:** 2021-06-28

**Authors:** Qi Wang, Zhen Lin, Hairong Chen, Tianyi Ma, Biyun Pan

**Affiliations:** ^1^Department of General Practice, Central South University Xiangya School of Medicine Affiliated Haikou Hospital, Haikou, China; ^2^Department of Geriatrics, Central South University Xiangya School of Medicine Affiliated Haikou Hospital, Haikou, China; ^3^Department of Cardiovasology, Central South University Xiangya School of Medicine Affiliated Haikou Hospital, Haikou, China

**Keywords:** coronary heart disease, predisposition, CYP2R1 variants, haplotype, lifestyle

## Abstract

**Propose:** Cytochrome P450 family 2 subfamily R member 1 (*CYP2R1*) variations can affect the activity of 25-hydroxylase, resulting in the deficiency of 25(OH)D, which leads to an increased incidence and mortality of coronary heart disease (CHD). The purpose is to assess the influence of *CYP2R1* variants on CHD risk among the Chinese Han population.

**Methods:** A total of 508 CHD patients and 510 healthy controls were enrolled. The MassARRAY platform completed genotyping of CYP2R1 variants. Odds ratios (ORs) with 95% confidence intervals (CI) were calculated using logistic regression analysis.

**Results:** Rs6486205 (OR = 1.25, 95% CI: 1.05–1.50, *p* = 0.014), rs10741657 (OR = 1.29, 95% CI: 1.08–1.54, *p* = 0.005), and rs2060793 (OR = 1.27, 95% CI: 1.06–1.51, *p* = 0.009) were associated with the increased susceptibility to CHD in the whole subjects. Interestingly, the relationships between these variants and CHD risk were observed in the subjects with age >60 years, males or non-smoker. Additionally, the haplotypes A_rs10741657_A_rs2060793_ and G_rs10741657_G_rs2060793_ had the higher risk of CHD, and the combination (rs6486205 and rs10741657) was the best multi-locus model.

**Conclusion:** Our study suggested the contribution of *CYP2R1* polymorphisms to the increased CHD predisposition in the Chinese Han population. Furthermore, the risk association was related to confounding factors for CHD, including age, sex, and smoking. These findings might help to strengthen the understanding of the *CYP2R1* gene in the occurrence of CHD.

## Introduction

Cardiovascular diseases (CVDs) cause 17.9 million deaths every year, accounting for a 31% global death toll, and approximately 85% of all CVD deaths are caused by coronary heart disease (CHD) and stroke (1). According to the 2018 China CVD report, there were approximately 290 million CVD patients in China, of which 11 million suffered from CHD (2). The incidence of CHD in women is lower than that of men, but the outcomes of CHD in females are worse than that of males (3). Coronary heart disease is one of the most common CVDs, characterized by remodeling and narrowing of coronary arteries (4). Coronary heart disease is a complex multifactorial disease. Previous studies have identified various risk factors for CHD, including smoking, drinking, hypertension, diabetes, dyslipidemia, and dietary factors (5, 6). To date, some genome-wide association studies have reported many susceptibility genes to CHD predisposition (7, 8), suggesting that genetic variants may have a central role in the occurrence of CHD.

The human cytochrome P450 family 2 subfamily R member 1 (*CYP2R1*) gene, located at 11p15.2, encodes a member of the CYP450 enzyme superfamily. CYP2R1 enzyme, produced in hepatic microsomes, is a physiologically important vitamin D hydroxylase that can convert vitamin D into 25-hydroxyvitamin D [25(OH)D] (9). Mutations in *CYP2R1* were related to the insufficient levels of 25(OH)D in individuals (10). The decreased level of vitamin D in circulation was related to the higher relative risk of CVDs, and the deficiency of vitamin D increased the mortality rate of CVDs (11, 12). Serum 25(OH)D in patients with coronary artery disease was correlated with cardiac structure and function (13). A large observational study suggested a reverse J-shaped relationship between serum 25(OH)D levels and CVDs, with the highest risk at lower levels (14). In previous observational research, low 25(OH)D might have a higher CHD risk, and this relationship might vary by race (15). These studies supported the physiological importance of *the CYP2R1* gene in the occurrence and development of CHD. Previously, genetic polymorphisms in *CYP2R1* were associated with various CVD-related diseases, including myocardial infarction and stroke, type 2 diabetes, and hypertension (16–19). However, the contribution of *CYP2R1* variants to CHD predisposition has not been reported among the Chinese Han population. These studies suggest that *CYP2R1* has a significant role in the development of CHD, which led us to propose the hypothesis that *CYP2R1* polymorphisms could be of importance in CHD susceptibility among the Chinese Han population.

*CYP2R1* rs10741657 and rs2060793 are involved in the regulation of gene expression and activity of 25-hydroxylase (20, 21), and the function of rs6486205 has not been reported to date. Here, three single nucleotide polymorphisms (SNPs) in *CYP2R1* (rs6486205, rs10741657, and rs2060793) were randomly selected and genotyped to assess the effect of single variants and combined SNPs on CHD predisposition among the Chinese Han population. Considering that age, sex, smoking, drinking, diabetes, and hypertension were confounding factors for CHD, stratification analysis was also performed to evaluate the contribution of *CYP2R1* SNPs to CHD risk.

## Materials and Methods

### Study Participants

A total of 1,018 genetically unrelated participants comprised 508 CHD patients, and 510 healthy controls were enrolled from Haikou People's Hospital. Patients were diagnosed with angiographically documented CHD by severe coronary stenosis (≥50%) in the main coronary arteries or their major branches. Patients with concomitant cardiomyopathy, congenital or valvar heart disease, brain, renal, liver, and lung disease, and tumor were excluded. Controls were recruited at the health examination of the hospital. No chest symptoms or electrocardiogram abnormalities confirmed healthy individuals without CHD. The controls were free from cerebrovascular disease, CVDs, peripheral vascular disease, kidney disease, autoimmune diseases, and cancer. All participants were Chinese Han population. Demographic information and clinical data were collected by standardized questionnaires and medical records, including age, sex, cigarette smoking, drinking, hypertension and diabetic status, blood biochemical index, etc. The Ethics Committee of Haikou People's Hospital (2018-179) approved the study, and informed consent was gained from all subjects. The study was carried out in compliance with the declaration of Helsinki.

### Genotyping

Peripheral blood samples (5 ml) were gathered in ethylenediaminetetraacetic acid tubes. Genomic DNA was isolated using commercial GoldMag DNA extraction kits (GoldMag, Xi′an, China). Three candidate SNPs in *CYP2R1* (rs6486205, rs10741657, and rs2060793) were randomly selected based on the minor allele frequency >0.05 from 1,000 Genomes Project database, Hardy–Weinberg equilibrium (HWE) >0.05, and the call rate >95%. Genotyping of *CYP2R1* polymorphisms was performed by the Agena MassARRAY platform (Agena, San Diego, CA, USA). Primers design and data management were carried out by supporting software. The primers were listed in Supplementary Table 1. Approximately 10% of the samples were randomly re-genotyped for quality control, and the concordance rate was 100%.

### Data Analysis

The distribution of characteristics between CHD patients and healthy controls were compared by χ^2^-test and sample *t*-test or Mann–Whitney U-test. The goodness of fit χ^2^-test analyzed HWE in controls and cases. Multiple genetic models were used to assess the contribution of *CYP2R1* variants to CHD susceptibility. Odds ratios (ORs) with 95% confidence intervals (CIs) were calculated using logistic regression analysis. We used Power and Sample Size Calculation software (http://sampsize.sourceforge.net/iface/s3.html#ccp) to calculate the power values. The Haploview v4.2 program constructed linkage disequilibrium and haplotype. Multifactor dimension reduction (MDR) was used to assess the best models for the SNP–SNP interaction and the gene–environment interaction on CHD risk. Analysis of variance was used to evaluate the association between genotypes of *CYP2R1* variants and blood biochemical index. Statistical analysis was completed by SPSS 20.0 and PLINK 1.0.7software. Two-tailed *p* < 0.05 was considered statistically significant.

## Results

### Features of Participants

The appearances of participants are shown in Table 1. The study included 508 CHD patients (62.2 ± 10.3 years, 334 males and 174 females) and 510 healthy controls (61.1 ± 9.0 49 years, 336 males and 174 females). There was no significant difference in age and sex distribution (*p* = 0.084 and 0.946, respectively) between the two groups. However, significant differences in smoking, alcohol consumption, and the concentration of leukocyte, red blood cell, platelet, hemoglobin, total cholesterol, high-density lipoprotein cholesterol (HDL-C), low-density lipoprotein cholesterol, triglyceride, Apo A1, and fasting blood glucose were found between the two groups (*p* < 0.05).

**Table 1:** Characteristics of patients with CHD and controls.

**Variable**	**Cases (*n* = 508)**	**Controls (*n* = 510)**	***p***
Age (year, mean ±*SD*)	62.2 ± 10.3	61.1 ± 9.0	0.084
>60/≤60	282/226	284/226	
Sex			0.946
Male/Female	334/174	336/174	
Smoking			** <0.001**
Yes/No	231/186	115/167	
Missing	91	228	
Alcohol consumption			** <0.001**
Yes/No	52/306	124/135	
Missing	150	251	
CHD with hypertension			
Yes/No	362/146		
CHD with diabetes			
Yes/No	190/318		
Leukocyte (109/L, IQR)	6.58 (2.33)	5.63 (2.08)	** <0.001**
RBC (109/L, IQR)	4.47 (0.69)	4.80 (0.59)	** <0.001**
Platelet (109/L, IQR)	189.00 (77.00)	211.00 (72.00)	** <0.001**
Hemoglobin (g/L, IQR)	137.00 (22.00)	149.00 (20.00)	** <0.001**
Total cholesterol (mmol/L, IQR)	4.00 (1.42)	4.74 (1.20)	** <0.001**
HDL-C (mmol/L, IQR)	1.08 (0.34)	1.11 (0.29)	**0.015**
LDL-C (mmol/L, IQR)	2.32 (1.22)	2.59 (0.91)	** <0.001**
Triglyceride (mmol/L, IQR)	1.36 (0.92)	1.49 (0.99)	** <0.001**
Apo A1 (g/L, IQR)	1.16 (0.35)	1.33 (0.34)	** <0.001**
FBG (mmol/L, IQR)	4.95 (1.63)	5.64 (0.81)	** <0.001**

*CHD, coronary heart disease; SD, standard deviation; IQR, interquartile range; RBC, red blood cell; HDL-C, high-density lipoprotein cholesterol; LDL-C, low-density lipoprotein cholesterol; Apo, apolipoprotein; FBG, fasting blood glucose. p-values were calculated by χ^2^-test or Mann–Whitney U-test for continuous variables and the Student's t-test for categorical variables. Bold indicates that p < 0.05 indicates statistical significance*.

### Association Between Cytochrome P450 Family 2 Subfamily R Member 1 Single Nucleotide Polymorphisms and Coronary Heart Disease Predisposition

Three *CYP2R1* SNPs were in line with HWE (all *p* > 0.05, Table 2). The frequencies of rs6486205-T, rs10741657-A, and rs2060793-A alleles were higher in the cases and were related to the higher risk of CHD (rs6486205, OR = 1.25, 95% CI: 1.05–1.50, *p* = 0.014; rs10741657, OR = 1.29, 95% CI: 1.08–1.54, *p* = 0.005; and rs2060793, OR = 1.27, 95% CI: 1.06–1.51, *p* = 0.009).

**Table 2:** Information about *CYP2R1* SNPs and association with CHD risk in allele model.

**SNPs ID**	**Chr:Position**	**Alleles**	**Frequency (MAF)**		**Location**	***p*****-value for HWE**		**Call rate (%)**	**OR (95% CI)**	***p***
		**(Minor/Major)**	**Case**	**Control**		**Control**	**Case**			
rs6486205	11:14859710	T/G	0.426	0.372	Intronic	0.776	0.120	99.5	1.25 (1.05–1.50)	**0.014**
rs10741657	11:14893332	A/G	0.424	0.363	5′-UTR	1.000	0.120	99.8	1.29 (1.08–1.54)	**0.005**
rs2060793	11:14893764	A/G	0.427	0.371	Promoter	0.850	0.085	99.8	1.27 (1.06–1.51)	**0.009**

*SNP, single nucleotide polymorphism; CHD, coronary heart disease; MAF, minor allele frequency; O(HET), Observed heterozygotes; E(HET), Expected heterozygotes; HWE, Hardy–Weinberg equilibrium. Bold indicates that p < 0.05 means data are statistically significant*.

Multiple genetic models analysis also displayed that rs6486205, rs10741657, and rs2060793 were associated with increased susceptibility to CHD (Table 3). Concretely, the risk association between rs6486205 and CHD occurrence was found under codominant (OR = 1.59, 95% CI: 1.11–2.29, *p* = 0.013, power = 79.74%), recessive (OR = 1.49, 95% CI: 1.07–2.08, *p* = 0.019, power = 65.92%), and additive (OR = 1.24, 95% CI: 1.04–1.47, *p* = 0.018). Rs10741657 increased the risk of CHD (AA vs. GG, OR = 1.72, 95% CI: 1.19–2.50, *p* = 0.004, power = 89.76%; AA vs. GG-GA, OR = 1.62, 95% CI: 1.15–2.27, *p* = 0.005, power = 80.90%; and additive, OR = 1.27, 95% CI: 1.07–1.52, *p* = 0.007). In addition, the higher CHD risk was observed for rs2060793 under the codominant (OR = 1.65, 95% CI: 1.14–2.37, *p* = 0.007, power = 85.40%), recessive (OR = 1.55, 95% CI: 1.11–2.17, *p* = 0.009, power = 74.00%), and additive models (OR = 1.25, 95% CI: 1.05–1.49, *p* = 0.013).

**Table 3:** Association between *CYP2R1* polymorphisms and CHD risk.

**SNP ID**	**Model**	**Genotype**	**Control**	**Case**	**Crude analysis**		**Adjusted by age and sex**		**AIC**	**BIC**
					**OR (95% CI)**	***p*-value**	**OR (95% CI)**	***p*-value**		
rs6486205	Codominant	GG	202	175	1		1			
		GT	234	230	1.14 (0.86–1.49)	0.363	1.13 (0.86–1.48)	0.391	1398.7	1413.4
		TT	72	100	1.60 (1.11–2.31)	**0.011**	1.59 (1.11–2.29)	**0.013**		
	Dominant	GG	202	175	1		1			
		GT-TT	306	330	1.25 (0.96–1.61)	0.093	1.24 (0.96–1.60)	0.104	1400.0	1409.8
	Recessive	GG-GT	436	405	1		1			
		TT	72	100	1.50 (1.07–2.08)	**0.017**	1.49 (1.07–2.08)	**0.019**	1397.6	1407.4
	Log-additive				1.24 (1.04–1.48)	**0.016**	1.24 (1.04–1.47)	**0.018**	1397.2	1407.1
rs10741657	Codominant	GG	206	177	1		1			
		GA	236	230	1.13 (0.87–1.49)	0.362	1.13 (0.86–1.48)	0.389	1396.3	1411.1
		AA	67	100	1.74 (1.20–2.51)	**0.003**	1.72 (1.19–2.50)	**0.004**		
	Dominant	GG	206	177	1		1			
		GA-AA	303	330	1.27 (0.98–1.64)	0.068	1.26 (0.98–1.62)	0.077	1399.3	1409.1
	Recessive	GG-GA	442	407	1		1			
		AA	67	100	1.62 (1.16–2.27)	**0.005**	1.62 (1.15–2.27)	**0.005**	1395.3	1405.1
	Log-additive				1.28 (1.07–1.52)	**0.006**	1.27 (1.07–1.52)	**0.007**	1395.4	1405.2
rs2060793	Codominant	GG	203	176	1		1			
		GA	236	229	1.12 (0.85–1.47)	0.417	1.11 (0.85–1.46)	0.451	1398.0	1414.7
		AA	71	102	1.66 (1.15–2.39)	**0.007**	1.65 (1.14–2.37)	**0.007**		
	Dominant	GG	203	176	1		1			
		GA-AA	307	331	1.24 (0.96–1.60)	0.093	1.23 (0.96–1.59)	0.106	1400.0	1409.8
	Recessive	GG-GA	439	405	1		1			
		AA	71	102	1.56 (1.12–2.17)	**0.009**	1.55 (1.11–2.17)	**0.009**	1396.8	1406.6
	Log-additive				1.25 (1.05–1.49)	**0.011**	1.25 (1.05–1.49)	**0.013**	1396.8	1406.6

*CHD, coronary heart disease; SNP, single nucleotide polymorphism; OR, odds ratio; 95% CI, 95% confidence interval; AIC, Akaike information criterion; BIC, Bayesian information criterion. p-values were calculated using logistic regression analysis adjusted by sex and age. Bold indicates that p < 0.05 means data are statistically significant*.

### Stratification Analysis for the Contribution of Cytochrome P450 Family 2 Subfamily R Member 1 Single Nucleotide Polymorphisms to Coronary Heart Disease Risk

Considering that age, sex, smoking, drinking, diabetes, and hypertension were confounding factors for CHD, stratification analyses were carried out to estimate the relation between *CYP2R1* SNPs and CHD risk.

Stratified by age, rs6486205, rs10741657, and rs2060793 increased the risk of CHD in the subjects with age >60 years. Significant results were shown under the allele, codominant, recessive, and additive models, as shown in Table 4. In sex stratification, the association between *CYP2R1* SNPs (rs6486205, rs10741657, and rs2060793) and CHD risk was observed in males but not in females (Table 4). For rs6486205, T allele and TT genotype carriers had a higher risk of CHD in males. For rs10741657, increased predisposition of CHD was found in the allele, codominant, dominant, and additive models. For rs2060793, A allele and AA genotype frequency distribution also differed between the cases and the controls among males.

**Table 4:** Association between *CYP2R1* polymorphisms and CHD risk according to stratification by age and sex.

**SNP ID**	**Model**	**Genotype**	**Control**	**Case**	**OR (95% CI)**	***p*-value**	**Control**	**Case**	**OR (95% CI)**	***p*-value**
**Age, years**			**>60**				**≤60**			
rs6486205	Allele	G	354	308	1		284	272	1	
		T	212	254	1.38 (1.09–1.75)	0.008	166	176	1.11 (0.85–1.45)	0.460
	Codominant	GG	107	89	1		95	86	1	
		GT	140	130	1.15 (0.79–1.67)	0.475	94	100	1.18 (0.78–1.77)	0.435
		TT	36	62	2.11 (1.28–3.49)	**0.004**	36	38	1.17 (0.68–2.00)	0.578
	Dominant	GG	107	89	1		95	86	1	
		GT-TT	176	192	1.35 (0.95–1.92)	0.099	130	138	1.17 (0.80–1.71)	0.408
	Recessive	GG-GT	247	219	1		189	186	1	
		TT	36	62	1.95 (1.24–3.07)	**0.004**	36	38	1.07 (0.65–1.77)	0.783
	Log-additive				1.39 (1.09–1.77)	**0.007**			1.10 (0.85–1.42)	0.478
rs10741657	Allele	G	361	310	1		287	274	1	
		A	205	254	1.44 (1.14–1.83)	0.003	165	176	1.12 (0.85–1.46)	0.420
	Codominant	GG	110	90	1		96	87	1	
		GA	141	130	1.16 (0.80–1.68)	0.438	95	100	1.16 (0.78–1.74)	0.468
		AA	32	62	2.40 (1.43–4.02)	**0.001**	35	38	1.20 (0.70–2.06)	0.515
	Dominant	GG	110	90	1		96	87	1	
		GA-AA	173	192	1.39 (0.98–1.98)	0.065	130	138	1.17 (0.80–1.71)	0.410
	Recessive	GG-GA	251	220	1		191	187	1	
		AA	32	62	2.20 (1.38–3.52)	**0.001**	35	38	1.11 (0.67–1.83)	0.686
	Log-additive				1.46 (1.15–1.87)	**0.002**			1.11 (0.86–1.44)	0.438
rs2060793	Allele	G	356	307	1		286	274	1	
		A	212	255	1.40 (1.10–1.77)	0.006	166	178	1.12 (0.86–1.46)	0.411
	Codominant	GG	107	89	1		96	87	1	
		GA	142	129	1.12 (0.77–1.63)	0.548	94	100	1.17 (0.78–1.76)	0.437
		AA	35	63	2.22 (1.34–3.68)	**0.002**	36	39	1.20 (0.70–2.05)	0.516
	Dominant	GG	107	89	1		96	87	1	
		GA-AA	177	192	1.34 (0.94–1.91)	0.104	130	139	1.18 (0.81–1.72)	0.388
	Recessive	GG-GA	249	218	1		190	187	1	
		AA	35	63	2.07 (1.31–3.27)	**0.002**	36	39	1.10 (0.67–1.81)	0.705
	Log-additive				1.41 (1.11–1.80)	**0.005**			1.11 (0.86–1.44)	0.431
**Sex**			**Males**				**Females**			
rs6486205	Allele	G	421	377	1		217	203	1	
		T	247	285	1.29 (1.03–1.61)	**0.024**	131	145	1.18 (0.87–1.60)	0.278
	Codominant	GG	132	111	1		70	64	1	
		GT	157	155	1.17 (0.84–1.64)	0.358	77	75	1.05 (0.65–1.67)	0.848
		TT	45	65	1.71 (1.08–2.69)	**0.022**	27	35	1.48 (0.80–2.73)	0.207
	Dominant	GG	132	111	1		70	64	1	
		GT-TT	202	220	1.29 (0.94–1.77)	0.116	104	110	1.16 (0.75–1.79)	0.511
	Recessive	GG-GT	289	266	1		147	139	1	
		TT	45	65	1.56 (1.03–2.36)	**0.036**	27	35	1.45 (0.83–2.53)	0.195
	Log-additive				1.28 (1.03–1.59)	**0.028**			1.19 (0.88–1.59)	0.258
rs10741657	Allele	G	426	381	1		222	203	1	
		A	244	285	1.31 (1.05–1.63)	**0.017**	126	145	1.26 (0.93–1.71)	0.140
	Codominant	GG	134	113	1		72	64	1	
		GA	158	155	1.16 (0.83–1.62)	0.384	78	75	1.07 (0.67–1.70)	0.793
		AA	43	65	1.78 (1.12–2.82)	**0.014**	24	35	1.73 (0.93–3.24)	0.086
	Dominant	GG	134	113	1		72	64	1	
		GA-AA	201	220	1.29 (0.94–1.77)	0.111	102	110	1.22 (0.79–1.88)	0.376
	Recessive	GG-GA	292	268	1		150	139	1	
		AA	43	65	1.64 (1.08–2.49)	**0.022**	24	35	1.67 (0.94–2.98)	0.079
	Log-additive				1.30 (1.04–1.61)	**0.021**			1.26 (0.94–1.70)	0.127
rs2060793	Allele	G	426	378	1		216	203	1	
		A	246	288	1.32 (1.06–1.64)	**0.013**	132	145	1.17 (0.86–1.58)	0.314
	Codominant	GG	134	112	1		69	64	1	
		GA	158	154	1.16 (0.83–1.63)	0.377	78	75	1.02 (0.64–1.63)	0.946
		AA	44	67	1.81 (1.15–2.86)	**0.011**	27	35	1.46 (0.79–2.69)	0.226
	Dominant	GG	134	112	1		69	64	1	
		GA-AA	202	221	1.30 (0.95–1.79)	0.100	105	110	1.13 (0.73–1.75)	0.589
	Recessive	GG-GA	292	266	1		147	139	1	
		AA	44	67	1.66 (1.10–2.52)	**0.016**	27	35	1.45 (0.83–2.53)	0.195
	Log-additive				1.31 (1.05–1.63)	**0.016**			1.17 (0.87–1.57)	0.292

*CHD, coronary heart disease; SNP, single nucleotide polymorphism; OR, odds ratio; 95% CI, 95% confidence interval. p-values were calculated using logistic regression analysis adjusted by sex and age. Bold indicates that p < 0.05 means data are statistically significant*.

Stratified by smoking (Table 5), rs2060793 A allele had a higher risk of CHD among smokers. Interestingly, three *CYP2R1* SNPs increased the susceptibility to CHD in non-smokers under the allele, codominant, dominant, and additive models. However, there was no significant association in drinking-stratified analysis, as shown in Supplementary Table 2.

**Table 5:** Association between *CYP2R1* polymorphisms and CHD risk according to stratification by smoking.

**SNP ID**	**Model**	**Genotype**	**Smoker**				**Non-smoker**			
			**Control**	**Case**	**OR (95% CI)**	***p*-value**	**Control**	**Case**	**OR (95% CI)**	***p*-value**
rs6486205	Allele	G	147	261	1		214	208	1	
		T	79	195	1.39 (1.00–1.94)	0.050	120	164	1.41 (1.04–1.90)	**0.027**
	Codominant	GG	50	80	1		71	59	1	
		GT	47	101	1.33 (0.81–2.18)	0.259	72	90	1.59 (0.99–2.56)	0.055
		TT	16	47	1.83 (0.94–3.58)	0.077	24	37	1.94 (1.03–3.64)	**0.040**
	Dominant	GG	50	80	1		71	59	1	
		GT-TT	63	147	1.46 (0.92–2.31)	0.109	96	127	1.68 (1.08–2.63)	**0.023**
	Recessive	GG-GT	97	181	1		143	149	1	
		TT	16	47	1.58 (0.85–2.94)	0.150	24	37	1.49 (0.84–2.65)	0.168
	Log-additive				1.35 (0.98–1.85)	0.066			1.43 (1.05–1.94)	**0.023**
rs10741657	Allele	G	150	267	1		215	206	1	
		A	80	193	1.36 (0.98–1.88)	0.069	119	166	1.46 (1.08–1.97)	**0.015**
	Codominant	GG	51	83	1		70	58	1	
		GA	48	101	1.28 (0.79–2.10)	0.320	75	90	1.54 (0.96–2.47)	0.076
		AA	16	46	1.77 (0.90–3.45)	0.096	22	38	2.18 (1.15–4.14)	**0.017**
	Dominant	GG	51	83	1		70	28	1	
		GA-AA	64	147	1.40 (0.89–2.22)	0.146	97	128	1.68 (1.08–2.63)	**0.023**
	Recessive	GG-GA	99	184	1		145	148	1	
		AA	16	46	1.55 (0.83–2.90)	0.165	22	38	1.71 (0.95–3.05)	0.071
	Log-additive				1.32 (0.96–1.81)	0.086			1.49 (1.09–2.03)	**0.012**
rs2060793	Allele	G	150	264	1		213	206	1	
		A	80	196	1.39 (1.00–1.93)	**0.048**	121	166	1.42 (1.05–1.92)	**0.023**
	Codominant	GG	51	82	1		70	58	1	
		GA	48	100	1.29 (0.79–2.10)	0.317	73	90	1.57 (0.98–2.53)	0.063
		AA	16	48	1.87 (0.96–3.64)	0.067	24	38	2.01 (1.07–3.77)	**0.031**
	Dominant	GG	51	82	1		70	58	1	
		GA-AA	64	148	1.43 (0.91–2.26)	0.125	97	128	1.68 (1.07–2.63)	**0.023**
	Recessive	GG-GA	99	182	1		143	148	1	
		AA	16	48	1.64 (0.88–3.05)	0.118	24	38	1.55 (0.88–2.75)	0.129
	Log-additive				1.35 (0.98–1.85)	0.063			1.44 (1.06–1.96)	**0.019**

*CHD, coronary heart disease; SNP, single nucleotide polymorphism; OR, odds ratio; 95% CI, 95% confidence interval. p-values were calculated using logistic regression analysis adjusted by sex and age; Bold indicates that p < 0.05 means data are statistically significant*.

Furthermore, the combined effect of *CYP2R1* SNPs on CHD patients with diabetes or hypertension was also assessed. However, *CYP2R1* SNPs were not significantly related to diabetes or hypertension in CHD patients (Supplementary Table 3).

### Haplotype and Multifactor Dimension Reduction Analysis for the Association Between Cytochrome P450 Family 2 Subfamily R Member 1 Single Nucleotide Polymorphisms and Coronary Heart Disease Risk

**Figure 1:**
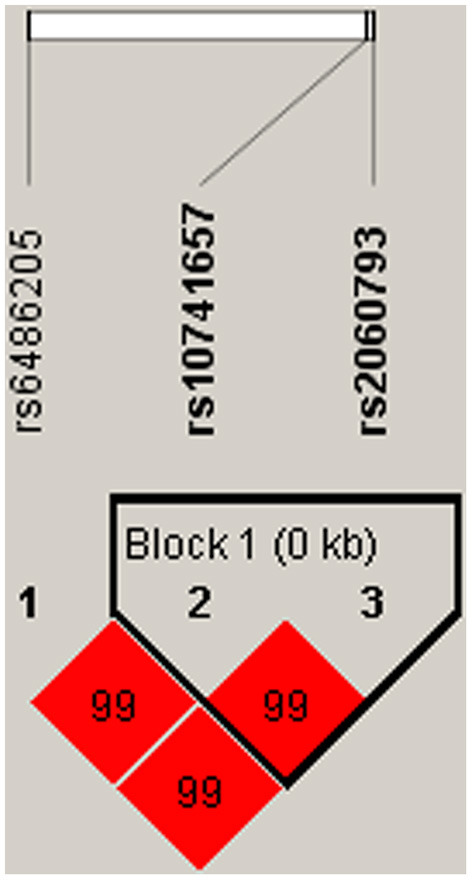
Haplotype block map for three SNPs in *CYP2R1*. A block was comprised of rs10741657 and rs2060793. Number in diamond represents *D*′-value.

Linkage disequilibrium analysis displayed that two SNPs (rs10741657 and rs2060793) in *CYP2R1* had strong linkage (Figure 1). Furthermore, the haplotypes A_rs10741657_A_rs2060793_ (OR = 1.29, 95% CI: 1.08–1.54, *p* = 0.005) and G_rs10741657_G_rs2060793_ (OR = 1.23, 95% CI: 1.04–1.47, *p* = 0.019) increased the predisposition of CHD (Table 6).

**Table 6:** Haplotype frequencies in *CYP2R1* and their associations with CHD risk.

**SNP**	**Haplotype**	**Frequency**		**χ^**2**^**	***p for χ*^**2**^**	**Crude analysis**		**Adjusted by age and sex**	
		**Case**	**Control**			**OR (95% CI)**	***p***	**OR (95% CI)**	***p***
rs10741657|rs2060793	AA	0.425	0.362	8.553	**0.003**	1.29 (1.09–1.54)	**0.004**	1.29 (1.08–1.54)	**0.005**
rs10741657|rs2060793	GG	0.426	0.372	6.078	**0.014**	1.24 (1.04–1.48)	**0.016**	1.23 (1.04–1.47)	**0.019**

*CHD, coronary heart disease; SNP, single nucleotide polymorphism; OR, odds ratio; 95% CI, 95% confidence interval. p-values were calculated using logistic regression analysis adjusted by sex and age. Bold indicates that p < 0.05 means data are statistically significant*.

Multifactor dimension reduction analysis of SNP–SNP interaction was performed to assess SNP interaction and its relation to CHD risk (Table 7). Rs10741657 was the best single-locus model (testing accuracy = 0.5079), and the two-locus model (rs6486205 and rs10741657) was the best combination in the multi-locus model (testing accuracy = 0.5049). Supplementary Figure 1 revealed the additive effect between *CYP2R1* rs6486205-TT, rs10741657-AA, and rs2060793-AA on conferring risk toward CHD occurrence. Multifactor dimension reduction analysis of gene–environment interaction suggested that drinking was found to be the most important environmental factor affecting CHD susceptibility. In addition, the gene–environment interaction model, composed of rs6486205, smoking, drinking, and age, showed higher testing-balanced accuracy (0.6132) and cross-validation consistency (7/10), indicating that this interaction model was a candidate gene–environment model in our population. The result of the dendrogram (Supplementary Figure 2) exhibited a strong synergy effect of gene–environment interaction on CHD risk.

**Table 7:** MDR analysis for *CYP2R1* SNP–SNP interaction and *CYP2R1* gene–environment interaction with CHD risk.

**Model**	**Training Bal. Acc**.	**Testing Bal. Acc**.	**CVC**	***p***
***CYP2R1*** **SNP–SNP INTERACTION**				
rs10741657	0.5334	0.5079	9/10	**0.0054**
rs6486205 and rs10741657	0.5379	0.5049	9/10	**0.0026**
rs6486205, rs10741657, and rs2060793	0.5382	0.5020	10/10	**0.0027**
***CYP2R1*** **GENE–ENVIRONMENT INTERACTION**				
Drinking	0.6688	0.6688	10/10	** <0.001**
Drinking and age	0.7304	0.6464	8/10	** <0.001**
rs6486205, smoking, and age	0.7861	0.6111	4/10	** <0.001**
rs6486205, smoking, drinking, and age	0.8309	0.6132	7/10	** <0.001**
rs10741657, smoking, drinking, age, and sex	0.8639	0.5888	6/10	** <0.001**
rs6486205, rs10741657, smoking, drinking, age, and sex	0.8651	0.5858	10/10	** <0.001**
rs6486205, rs10741657, rs2060793, smoking, drinking, age, and sex	0.8651	0.5858	10/10	** <0.001**

*MDR, multifactor dimensionality reduction; Bal. Acc., balanced accuracy; CVC, cross-validation consistency; OR, odds ratio; CI, confidence interval. p-values were calculated using χ^2^-tests. Bold indicates that p < 0.05 indicates statistical significance*.

### Association Between Genotypes of Cytochrome P450 Family 2 Subfamily R Member 1 Variants and Blood Biochemical Index

Next, the association between *CYP2R1* SNPs and blood biochemical index in healthy control and CHD patients was assessed, as displayed in Table 8. We found that the genotypes of rs6486205 (*p* = 0.041), rs10741657 (*p* = 0.039), and rs2060793 (*p* = 0.031) were associated with serum concentration of HDL-C.

**Table 8:** Association of *CYP2R1* polymorphisms with clinical characteristics.

**Characteristics**	**rs6486205**							
	**Control**				**Case**			
	**TT**	**GT**	**GG**	***p***	**TT**	**GT**	**GG**	***p***
Leukocyte (10^9^/L, IQR)	5.88 (1.54)	5.75 (1.50)	5.85 (1.52)	0.756	6.74 (1.82)	6.72 (1.89)	7.09 (1.94)	0.147
RBC (10^9^/L, IQR)	4.78 (0.43)	4.82 (0.47)	4.83 (0.47)	0.782	4.46 (0.49)	4.44 (0.54)	4.52 (0.55)	0.368
Hemoglobin (g/L, IQR)	146.88 (13.14)	147.42 (15.35)	148.36 (14.6)	0.741	135.35 (15.80)	135.38 (16.62)	137.96 (16.57)	0.279
Platelet (10^9^/L, IQR)	216.66 (66.66)	212.18 (51.59)	211.55 (55.51)	0.823	190.46 (56.60)	194.58 (57.95)	200.98 (59.66)	0.349
Total cholesterol (mmol/L, IQR)	4.81 (0.78)	4.74 (0.82)	4.7 (0.97)	0.724	3.95 (1.02)	4.07 (1.06)	4.16 (1.05)	0.301
Triglyceride (mmol/L, IQR)	1.69 (0.74)	1.58 (0.69)	1.73 (0.77)	0.161	1.47 (0.66)	1.58 (0.81)	1.50 (0.72)	0.465
HDL-C (mmol/L, IQR)	1.12 (0.21)	1.17 (0.23)	1.13 (0.23)	0.188	1.09 (0.24)	1.09 (0.23)	1.15 (0.27)	**0.041**
LDL-C (mmol/L, IQR)	2.69 (0.71)	2.57 (0.64)	2.57 (0.75)	0.486	2.29 (0.84)	2.43 (0.85)	2.36 (0.84)	0.434
Apo A1 (g/L, IQR)	1.23 (0.18)	1.41 (0.21)	1.34 (0.24)	0.321	1.14 (0.21)	1.16 (0.24)	1.20 (0.25)	0.103
FBG (mmol/L, IQR)	5.90 (1.09)	5.85 (1.06)	6.03 (1.13)	0.313	5.56 (1.60)	5.38 (1.37)	5.52 (1.64)	0.660
**Characteristics**	**rs10741657**							
	**Control**				**Case**			
	**AA**	**GA**	**GG**	***p***	**AA**	**GA**	**GG**	***p***
Leukocyte (10^9^/L, IQR)	5.82 (1.50)	5.76 (1.51)	5.86 (1.52)	0.840	6.70 (1.84)	6.73 (1.88)	7.10 (1.93)	0.136
RBC (10^9^/L, IQR)	4.77 (0.43)	4.83 (0.47)	4.82 (0.47)	0.693	4.47 (0.51)	4.44 (0.54)	4.51 (0.54)	0.401
Hemoglobin (g/L, IQR)	146.96 (13.4)	147.53 (15.18)	148.13 (14.72)	0.855	135.97 (16.32)	135.46 (16.67)	137.97 (16.44)	0.337
Platelet (10^9^/L, IQR)	216.44 (67.12)	213.53 (51.58)	210.60 (55.59)	0.762	189.20 (55.44)	194.26 (58.26)	201.17 (58.99)	0.259
Total cholesterol (mmol/L, IQR)	4.81 (0.76)	4.75 (0.82)	4.69 (0.96)	0.608	3.94 (1.02)	4.08 (1.06)	4.15 (1.05)	0.281
Triglyceride (mmol/L, IQR)	1.67 (0.67)	1.59 (0.72)	1.72 (0.77)	0.218	1.47 (0.66)	1.58 (0.81)	1.51 (0.71)	0.437
HDL-C (mmol/L, IQR)	1.11 (0.19)	1.18 (0.23)	1.13 (0.24)	0.102	1.10 (0.24)	1.09 (0.23)	1.15 (0.27)	**0.039**
LDL-C (mmol/L, IQR)	2.72 (0.71)	2.58 (0.65)	2.56 (0.74)	0.346	2.28 (0.85)	2.43 (0.85)	2.36 (0.84)	0.324
Apo A1 (g/L, IQR)	1.23 (0.18)	1.37 (0.16)	1.34 (0.24)	0.446	1.14 (0.21)	1.16 (0.24)	1.20 (0.25)	0.125
FBG (mmol/L, IQR)	5.89 (1.13)	5.85 (1.06)	6.03 (1.13)	0.301	5.53 (1.59)	5.39 (1.38)	5.52 (1.63)	0.749
**Characteristics**	**rs2060793**							
	**Control**				**Case**			
	**AA**	**GA**	**GG**	***p***	**AA**	**GA**	**GG**	***p***
Leukocyte (10^9^/L, IQR)	5.81 (1.47)	5.77 (1.51)	5.85 (1.52)	0.882	6.71 (1.82)	6.73 (1.89)	7.09 (1.93)	0.152
RBC (10^9^/L, IQR)	4.78 (0.44)	4.82 (0.46)	4.83 (0.47)	0.823	4.47 (0.50)	4.44 (0.54)	4.51 (0.54)	0.402
Hemoglobin (g/L, IQR)	146.88 (13.26)	147.44 (15.27)	148.34 (14.64)	0.754	135.96 (16.18)	135.43 (16.71)	137.87 (16.44)	0.360
Platelet (10^9^/L, IQR)	217.62 (66.83)	212.17 (51.58)	211.26 (55.53)	0.745	190.37 (56.04)	194.29 (58.39)	201.57 (58.96)	0.284
Total cholesterol (mmol/L, IQR)	4.84 (0.75)	4.73 (0.83)	4.70 (0.97)	0.570	3.93 (1.01)	4.08 (1.07)	4.15 (1.05)	0.269
Triglyceride (mmol/L, IQR)	1.70 (0.74)	1.57 (0.69)	1.73 (0.77)	0.112	1.47 (0.66)	1.58 (0.81)	1.50 (0.72)	0.425
HDL-C (mmol/L, IQR)	1.13 (0.21)	1.17 (0.23)	1.13 (0.24)	0.200	1.10 (0.24)	1.09 (0.23)	1.15 (0.27)	**0.031**
LDL-C (mmol/L, IQR)	2.72 (0.69)	2.57 (0.65)	2.56 (0.75)	0.345	2.28 (0.84)	2.43 (0.85)	2.36 (0.84)	0.332
Apo A1 (g/L, IQR)	1.23 (0.18)	1.41 (0.21)	1.34 (0.24)	0.321	1.14 (0.21)	1.16 (0.24)	1.20 (0.25)	0.121
FBG (mmol/L, IQR)	5.89 (1.10)	5.85 (1.06)	6.03 (1.14)	0.297	5.55 (1.59)	5.38 (1.38)	5.52 (1.64)	0.667

*CHD, coronary heart disease; SD, standard deviation; IQR, interquartile range; RBC, red blood cell; HDL-C, high-density lipoprotein cholesterol; LDL-C, low-density lipoprotein cholesterol; Apo, apolipoprotein; FBG, fasting blood glucose. p-values were calculated by analysis of variance test. Bold indicates that p < 0.05 indicates statistical significance*.

## Discussion

In the study, we explored the contribution of three *CYP2R1* SNPs to CHD risk in the Chinese Han population. Our results showed that rs6486205, rs10741657, and rs2060793 increased the predisposition of CHD in the whole subjects. Interestingly, the relations between these SNPs and CHD risk were observed in the subjects with age >60 years, males, or non-smokers. Additionally, the haplotypes A_rs10741657_A_rs2060793_ and G_rs10741657_G_rs2060793_ had a higher risk of CHD, and the combination (rs6486205 and rs10741657) was the best multi-locus model. This is first to reveal the correlation between *CYP2R1* variants and CHD susceptibility in the Chinese Han population, and these variants could serve as potential biomarkers of CHD susceptibility.

Variation of *CYP2R1* can affect the activity of 25-hydroxylase, resulting in the deficiency of 25(OH)D, which in turn leads to an increasing incidence and mortality of CVDs (22). Rs10741657, located in the non-coding region 5′-untranslated region, can regulate gene expression and activity of 25-hydroxylase (20). *CYP2R1* rs10741657 leads to the lowered synthesis of CYP2R1 for the variant G-allele (23), presumably resulting in lowered conversion rate of cholecalciferol into 25(OH)D (20, 24). Rs10741657 was reported to be associated with type 2 diabetes, ischemic stroke, and blood pressure (18, 19, 25). *CYP2R1* rs2060793, in the promoter region, is involved in the regulation of gene transcription (21). Furthermore, rs2060793 was also reported to be associated with 25(OH)D concentrations (26). The association of rs2060793 with atrial fibrillation, gestational diabetes mellitus, and type 1 diabetes was reported (27–29). In the Egyptian population, rs10741657 and rs2060793 were related to 25(OH)D levels and might be novel genetic markers for CADs (30). Our study revealed that rs10741657 and rs2060793 increased the risk of CHD in the Chinese Han population, which was consistent with previous studies. We also found that rs6486205 contributed to CHD susceptibility. However, there was no study reporting rs6486205 and the relationship of rs6486205 to disease risk. Whether the SNPs identified are also recurrent in other diseases with *CYP2R1* mutations is necessary to explore.

Aging is a risk factor for CHD, and the potential risk factors for CHD incidence are influenced by age-related changes (31). In our study, the relationship between *CYP2R1* SNPs (rs6486205, rs10741657, and rs2060793) and the increased CHD risk was observed in the subjects with age >60 years. Moreover, sex difference was related to the adult mortality of CHD, which is greater mortality rates and risks in males than females (32). We also found the relationship between *CYP2R1* SNPs (rs6486205, rs10741657, and rs2060793) and the increased CHD risk in males. These results suggested that the association might be age- and sex-dependent. Previously, smoking is a significant risk factor for CHD, but polygenic risk scores have a better predictive effect among non-smokers compared with smokers (23). Our results displayed that *CYP2R1* SNPs contributed to the increased CHD predisposition in non-smokers. This is in line with previous evidence that genetic factors may have a more important role in CHD. Epidemiologic research has revealed that alcohol consumption is related to the risk of CHD incidence (33). Besides, diabetes and hypertension are the major risk factors for CHD incidence (34). However, no association was observed in drinkers and in CHD patients with diabetes or hypertension. Further studies are necessary to verify our results.

Coronary heart disease is a complex multifactorial disease. Multiple genetic and environmental risk factors contribute to CHD. We also investigated the association of combined SNPs in *CYP2R1* with CHD risk. The results showed that the haplotypes A_rs10741657_A_rs2060793_ and G_rs10741657_G_rs2060793_ increased the predisposition of CHD. SNP–SNP interaction analysis displayed the accumulated effect of *CYP2R1* variants on conferring CHD risk. Moreover, gene–environment interaction suggested that drinking was found to be the most important environmental factor affecting CHD susceptibility. In addition, the gene–environment interaction model composed rs6486205, smoking, drinking, and age, indicating that the combined effect of gene–environment interaction should be appreciated in the pathogenesis of CHD.

Previous studies have shown that HDL-C levels are considered independent risk factors for the development of CHD (35, 36). We found that the genotypes of rs6486205, rs10741657, and rs2060793 were associated with serum concentration of HDL-C, suggesting that *CYP2R1* polymorphisms might play an important role in serum concentration of HDL-C. However, more functional studies are required.

Several limitations should be acknowledged. First, based on hospital-based research, selection bias was inevitable. Here, age and sex were matched to reduce the bias. Second, the subjects were the Chinese Han population, so these results should be interpreted with caution. Further studies in other different ethnic populations are needed to confirm our finding. Third, only three variants in *CYP2R1* were assessed, and the risk association of other *CYP2R1* SNPs remains to be further investigated. Moreover, the potential impact of the SNPs on the protein function of CYP2R1 is unknown; therefore, additional studies will be required. Another, whether the SNPs identified are also recurrent in other diseases with *CYP2R1* mutations is necessary to explore. Four, the clinical symptoms, such as the severity of CHD, stage of CHD, were not examined. In the future, we would like to enlarge the sample size and complete the clinical symptoms, such as the severity of disease, stage of the disease to evaluate the association between *CYP2R1* SNPs and the clinical symptoms of CHD.

## Conclusion

In conclusion, our research firstly suggested the contribution of *CYP2R1* SNPs (rs6486205, rs10741657, and rs2060793) and haplotypes (A_rs10741657_A_rs2060793_ and G_rs10741657_G_rs2060793_) to the increased CHD predisposition among the Chinese Han population, and these variants could serve as potential biomarkers of CHD susceptibility. Furthermore, the risk association was related to confounding factors for CHD, including age, sex, and smoking. These findings might help to strengthen the understanding of the *CYP2R1* gene in the occurrence of CHD. Our finding increased our knowledge regarding the effect of the *CYP2R1* gene on the process of CHD and also provided some data for future explorations of the relationship between the *CYP2R1* gene and CHD risk in different populations.

## Data Availability Statement

The data presented in the study are deposited in the Zenodo repository: https://zenodo.org/record/4977934#.YMxSmWhKiUl.

## Ethics Statement

The study was approved by the Ethics Committee of the Haikou City people's Hospital, and informed consent was gained from all subjects. The patients/participants provided their written informed consent to participate in this study.

## Author Contributions

QW: writing and conceptualization. ZL and HC: methodology. TM and BP: data curation. All authors contributed to the article and approved the submitted version.

## Conflict of Interest

The authors declare that the research was conducted in the absence of any commercial or financial relationships that could be construed as a potential conflict of interest.
