# Supplementary material for: Effect of Cytochrome P450 Family 2 Subfamily R Member 1 Variants on the Predisposition of Coronary Heart Disease in the Chinese Han Population
Source: Front Cardiovasc Med. 2021 Jun 28;8:652729. doi: 10.3389/fcvm.2021.652729 (PMC8273490; doi:10.3389/fcvm.2021.652729)
Supplement: Supplementary file 1 [file Table_1.DOCX]

Suppl_**Table 1. Primers sequence of PCR and UEP for *CYP2R1* SNPs in this study**

| SNPs | First Primer(5'-3') | Second Primer (5'-3') | UEP_DIR | | | UEP SEQ (5'-3') |
| --- | --- | --- | --- | --- | --- | --- |
| rs6486205 | ACGTTGGATGCCTCACTGCACCTGTTATAC | ACGTTGGATGTCTCAAGTAGAAGAGGGTGC | | F | ATTCTTTAGCTGGCTAGT | |
| rs10741657 | ACGTTGGATGTAGCAGTTGATCTCAGCTCC | ACGTTGGATGTAGAAAACGCCTGGTGGTTG | | R | GACTTTCCTTGACAGCCCT | |
| rs2060793 | ACGTTGGATGTGCAGATGGAATTAAAGGGC | ACGTTGGATGGATTATATTGGGCCCACCTG | | R | ctcccATCAGCTGATCTTTGAGT | |

SNP: Single nucleotide polymorphism; UEP: Unextended mini sequencing primer; DIR, direction.

Suppl_Table 2. Association between *CYP2R1* polymorphisms and CHD risk according to the stratification by drinking

| SNP ID | Model | Genotype | Drinkers | | | | | Non-drinkers | | | |
| --- | --- | --- | --- | --- | --- | --- | --- | --- | --- | --- | --- |
|  |  |  | Control | Case | | OR (95% CI) | *p*-value | Control | Case | OR (95% CI) | *p*-value |
| rs6486205 | Allele | G | 161 | | 56 | 1 |  | 167 | 338 | 1 |  |
|  |  | T | 83 | | 46 | 1.59 (0.99–2.55) | 0.052 | 103 | 272 | 1.31 (0.97–1.75) | 0.075 |
|  | Codominant | GG | 55 | | 17 | 1 |  | 55 | 96 | 1 |  |
|  |  | GT | 51 | | 22 | 1.40 (0.67–2.94) | 0.376 | 57 | 146 | 1.42 (0.90–2.24) | 0.134 |
|  |  | TT | 16 | | 12 | 2.54 (1.00–6.48) | **0.050** | 23 | 63 | 1.48 (0.82–2.67) | 0.189 |
|  | Dominant | GG | 55 | | 17 | 1 |  | 55 | 96 | 1 |  |
|  |  | GT-TT | 67 | | 34 | 1.66 (0.84–3.31) | 0.146 | 80 | 209 | 1.44 (0.94–2.20) | 0.095 |
|  | Recessive | GG-GT | 106 | | 39 | 1 |  | 112 | 242 | 1 |  |
|  |  | TT | 16 | | 12 | 2.13 (0.92–4.96) | 0.078 | 23 | 63 | 1.22 (0.72–2.09) | 0.462 |
|  | Log-additive |  |  | |  | 1.56 (0.99–2.48) | 0.057 |  |  | 1.25 (0.94–1.67) | 0.131 |
| rs10741657 | Allele | G | 164 | | 58 | 1 |  | 168 | 340 | 1 |  |
|  |  | A | 84 | | 46 | 1.55 (0.97–2.47) | 0.066 | 102 | 272 | 1.32 (0.98–1.77) | 0.065 |
|  | Codominant | GG | 56 | | 19 | 1 |  | 54 | 97 | 1 |  |
|  |  | GA | 52 | | 22 | 1.31 (0.63–2.73) | 0.465 | 60 | 146 | 1.32 (0.84–2.07) | 0.235 |
|  |  | AA | 16 | | 12 | 2.44 (0.97–6.18) | 0.059 | 21 | 63 | 1.58 (0.87–2.89) | 0.136 |
|  | Dominant | GG | 56 | | 18 | 1 |  | 54 | 97 | 1 |  |
|  |  | GA-AA | 68 | | 34 | 1.57 (0.80–3.09) | 0.190 | 81 | 209 | 1.39 (0.91–2.12) | 0.133 |
|  | Recessive | GG-GA | 108 | | 40 | 1 |  | 114 | 243 | 1 |  |
|  |  | AA | 16 | | 12 | 2.12 (0.92–4.93) | 0.079 | 21 | 63 | 1.36 (0.78–2.35) | 0.278 |
|  | Log-additive |  |  | |  | 1.52 (0.96–2.40) | 0.071 |  |  | 1.27 (0.95–1.70) | 0.111 |
| rs2060793 | Allele | G | 164 | | 58 | 1 |  | 166 | 337 | 1 |  |
|  |  | A | 84 | | 46 | 1.55 (0.97–2.47) | 0.066 | 104 | 273 | 1.29 (0.97–1.73) | 0.085 |
|  | Codominant | GG | 56 | | 19 | 1 |  | 54 | 96 | 1 |  |
|  |  | GA | 52 | | 22 | 1.31 (0.63–2.73) | 0.465 | 58 | 145 | 1.37 (0.86–2.16) | 0.183 |
|  |  | AA | 16 | | 12 | 2.44 (0.97–6.18) | 0.059 | 23 | 64 | 1.48 (0.82–2.67) | 0.193 |
|  | Dominant | GG | 56 | | 18 | 1 |  | 54 | 96 | 1 |  |
|  |  | GA-AA | 68 | | 34 | 1.57 (0.80–3.09) | 0.190 | 81 | 209 | 1.40 (0.91–2.14) | 0.124 |
|  | Recessive | GG-GA | 108 | | 40 | 1 |  | 112 | 241 | 1 |  |
|  |  | AA | 16 | | 12 | 2.12 (0.92–4.93) | 0.079 | 23 | 64 | 1.24 (0.73–2.12) | 0.425 |
|  | Log-additive |  |  | |  | 1.52 (0.96–2.40) | 0.071 |  |  | 1.24 (0.93–1.66) | 0.145 |

SNP, single nucleotide polymorphism; CHD, coronary heart disease; OR, odds ratio; 95% CI, 95% confidence interval.

*p* values were calculated by logistic regression analysis with adjustments for age and gender.

Suppl_Table 3. Association of *CYP2R1* polymorphisms in CHD patients with diabetes or hypertension

| SNP ID | Model | Genotype | CHD with diabetes | | | | CHD with hypertension | | | |
| --- | --- | --- | --- | --- | --- | --- | --- | --- | --- | --- |
|  |  |  | Yes | No | OR (95% CI) | *p*-value | Yes | No | OR (95% CI) | *p*-value |
| rs6486205 | Allele | G | 416 | 164 | 1 |  | 224 | 356 | 1 |  |
|  |  | T | 306 | 124 | 1.03 (0.78–1.36) | 0.845 | 156 | 274 | 1.11 (0.85–1.43) | 0.448 |
|  | Codominant | GG | 124 | 51 | 1 |  | 68 | 107 | 1 |  |
|  |  | GT | 168 | 62 | 0.89 (0.58–1.38) | 0.611 | 88 | 142 | 1.03 (0.69–1.56) | 0.878 |
|  |  | TT | 69 | 31 | 1.07 (0.63–1.83) | 0.805 | 34 | 66 | 1.20 (0.71–2.02) | 0.489 |
|  | Dominant | GG | 124 | 51 | 1 |  | 68 | 107 | 1 |  |
|  |  | GT-TT | 237 | 93 | 0.94 (0.63–1.42) | 0.783 | 122 | 208 | 1.08 (0.74–1.59) | 0.692 |
|  | Recessive | GG-GT | 292 | 113 | 1 |  | 136 | 249 | 1 |  |
|  |  | TT | 69 | 31 | 1.14 (0.71–1.84) | 0.589 | 34 | 66 | 1.18 (0.74–1.88) | 0.486 |
|  | Log-additive |  |  |  | 1.02 (0.78–1.33) | 0.907 |  |  | 1.09 (0.84–1.40) | 0.519 |
| rs10741657 | Allele | G | 416 | 168 | 1 |  | 225 | 359 | 1 |  |
|  |  | A | 306 | 124 | 1.00 (0.76–1.32) | 0.981 | 155 | 275 | 1.11 (0.86–1.44) | 0.420 |
|  | Codominant | GG | 124 | 53 | 1 |  | 68 | 109 | 1 |  |
|  |  | GA | 168 | 62 | 0.86 (0.56–1.33) | 0.492 | 89 | 141 | 0.99 (0.66–1.50) | 0.976 |
|  |  | AA | 69 | 31 | 1.03 (0.60–1.76) | 0.917 | 33 | 67 | 1.23 (0.73–2.08) | 0.432 |
|  | Dominant | GG | 124 | 53 | 1 |  | 68 | 109 | 1 |  |
|  |  | GA-AA | 237 | 93 | 0.91 (0.61–1.36) | 0.640 | 122 | 208 | 1.06 (0.72–1.55) | 0.768 |
|  | Recessive | GG-GA | 292 | 115 | 1 |  | 157 | 250 | 1 |  |
|  |  | AA | 69 | 31 | 1.12 (0.70–1.81) | 0.641 | 33 | 67 | 1.24 (0.77–1.98) | 0.373 |
|  | Log-additive |  |  |  | 0.99 (0.76–1.30) | 0.960 |  |  | 1.09 (0.85–1.41) | 0.494 |
| rs2060793 | Allele | G | 415 | 166 | 1 |  | 224 | 357 | 1 |  |
|  |  | A | 309 | 124 | 1.00 (0.76–1.32) | 0.982 | 156 | 277 | 1.11 (0.86–1.44) | 0.411 |
|  | Codominant | GG | 124 | 52 | 1 |  | 68 | 108 | 1 |  |
|  |  | GA | 167 | 62 | 0.88 (0.57–1.36) | 0.568 | 88 | 141 | 1.02 (0.67–1.53) | 0.945 |
|  |  | AA | 71 | 31 | 1.02 (0.60–1.74) | 0.942 | 34 | 68 | 1.23 (0.73–2.07) | 0.433 |
|  | Dominant | GG | 124 | 52 | 1 |  | 68 | 108 | 1 |  |
|  |  | GA-AA | 238 | 93 | 0.92 (0.62–1.38) | 0.696 | 122 | 209 | 1.08 (0.73–1.58) | 0.710 |
|  | Recessive | GG-GA | 291 | 114 | 1 |  | 156 | 249 | 1 |  |
|  |  | AA | 71 | 31 | 1.10 (0.68–1.76) | 0.707 | 34 | 68 | 1.22 (0.77–1.94) | 0.400 |
|  | Log-additive |  |  |  | 0.99 (0.76–1.30) | 0.962 |  |  | 1.10 (0.85–1.41) | 0.479 |

SNP, single nucleotide polymorphism; CHD, coronary heart disease; hypertension, HYP; OR, odds ratio; 95% CI, 95% confidence interval.

*p* values were calculated by logistic regression analysis with adjustments for age and gender.
