# Supplementary figures and images for: Effect of Cytochrome P450 Family 2 Subfamily R Member 1 Variants on the Predisposition of Coronary Heart Disease in the Chinese Han Population
Source: Front Cardiovasc Med. 2021 Jun 28;8:652729. doi: 10.3389/fcvm.2021.652729 (PMC8273490; doi:10.3389/fcvm.2021.652729)

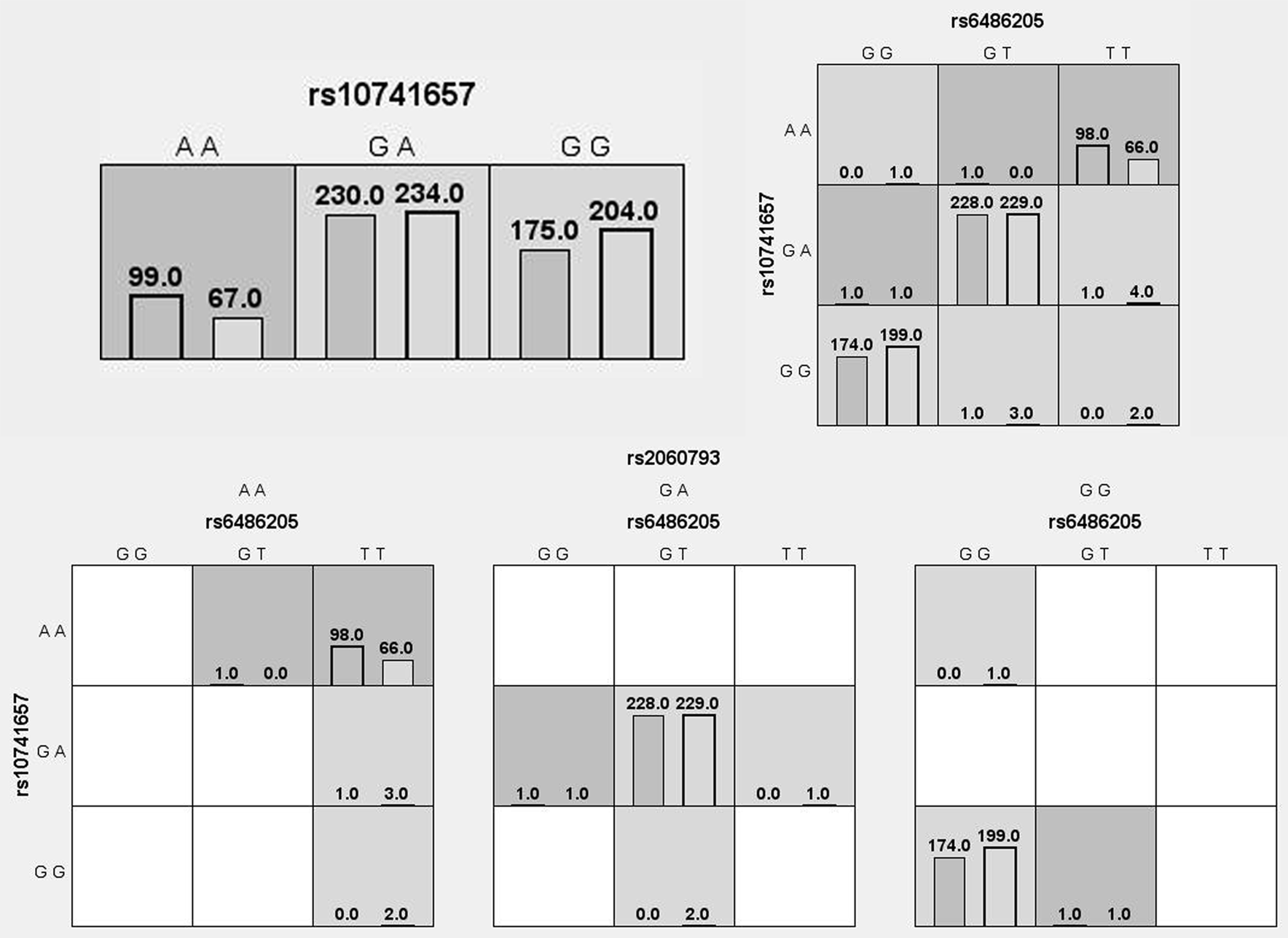

Supplement: Supplementary file 3 [file Image_1.TIF]

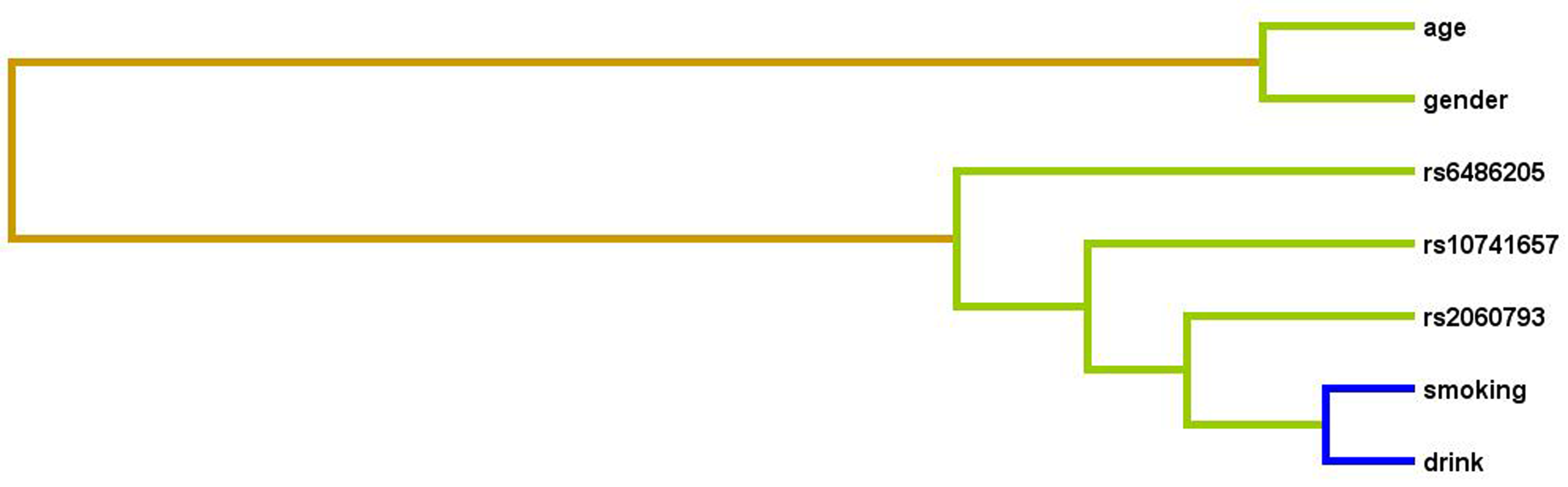

Supplement: Supplementary file 4 [file Image_2.TIF]
